# Regulatory void for vaccines targeting eukaryotic parasites of farmed and domestic animals and fish

**DOI:** 10.3389/fpara.2022.1073801

**Published:** 2022-12-05

**Authors:** Peter Holdsworth, Maggie Fisher

**Affiliations:** ^1^ PAH Consultancy Pty Ltd, Canberra, ACT, Australia; ^2^ Veterinary Research Management Limited, Malvern, United Kingdom

**Keywords:** parasite, regulation, efficacy, veterinary, vaccine, eukaryotic

## Introduction

Vaccines targeting eukaryotic parasites of domestic or native animals, or fish involve significant investments to reach the market, accompanied by substantial risk including uncertainty on regulatory requirements and financial returns.

Perhaps unsurprisingly, to date the number of these vaccines commercially available has been limited (**Table 1**). This is both unfortunate and frustrating as vaccine products could potentially:

- Treat established and/or prevent establishment of parasites in the host;
- Prevent/minimise environmental contamination with parasite life cycle stages;
- Support animal health and welfare;
- Prevent/minimise risk from zoonotic parasites;
- Provide a production tool to support profitable agriculture.

**Table 1:** Some commercially available vaccine technologies targeting eukaryotic parasites.

Primary vaccinated animal	Vaccine type	Target parasites
Poultry Turkey	Live virulent oocysts Attenuated Subunit of GAM 56 Live, nonattenuated	*Eimeria* spp. *Eimeria* spp. *Eimeria maxima* *Eimeria* spp.
Sheep Pig	Attenuated S-48 strain Subunit recombinant of 45W antigen of oncosphere protein Antigen derived from worm gut membrane proteins Single recombinant oncosphere antigen Recombinant subunit	*Toxoplasma gondii* *Taenia ovis* * Haemonchus* spp. *Echinococcus granulosus* Taenia *solium*
Cattle	Killed tachyzoites Attenuated cell line of ground up tick sporozoites Non attenuated live sporozoites Attenuated merozoites Live, nonpathogenic Live irradiated L3 larvae Recombinant Bm86 protein Inactivated parasite	*Neospora caninum* *Theleria annulata* *Theleria parva* *Babesia bovis; Babesia* *bigemina* *Besnoitia besnoiti* *Dictyocaulus viviparus;* *Dictyocaulus filaria* *Rhipicephalus microplus* *Trichomonas foetus*
Fish	Third generation synthetic peptide subunit	*Caligus rogercresseyi*
Dog	Subunit of soluble parasite antigen Killed trophozoites Recombinant protein A2 of amastigotes Purified excreted-secreted proteins Recombinant chimerical protein (Q)	*Babesia canis* *Giardia duodenalis* *Leishmania donovani* *Leishmania infantum* *Leishmania infantum*

Partly incorporating data from Sharma et al. (2015).

Vaccines could also be a valuable tool to aid in management of emergent insecticidal, acaricidal or anthelmintic resistance in parasite populations. Additionally, the availability and adoption of future commercially successful vaccines could aid in the reduction of chemicals (insecticide, acaricide) usage in general and specifically aid in management of the present concerns in some countries, of contamination of the environment from treatments with such product types (Little and Boxall, 2020; Loeb, 2020). Additionally, reduction of such chemical usage in production animals should assist in reducing the risk of illegal chemical residues in animal derived protein, hide and fiber.

## Present situation

Achieving effective vaccines against eukaryotic parasites is scientifically challenging. The present era of “omics” offers perhaps our most opportune period to date to utilize science to deliver on the need for vaccines targeting eukaryotic parasites of animals. Apart from the discovery phase of science, there is a need to understand regulatory requirements, at country and at regional level, to develop potential candidate vaccines to ultimate commercial products. Regulatory requirements invariably cover product quality; product safety to humans, animals and the environment; product efficacy and in some cases demonstration that the use of the product will not result in undue negative impacts on trade in animal derived protein, hide or fiber based on chemical residues (APVMA, 2022).

At present clear regulatory requirements relating to demonstration of efficacy of a new vaccine targeting eukaryotic parasites appear unclear. Traditional vaccine products targeting prokaryotic organisms have, in most parts, clearly defined regulatory requirements in developed countries. Regulatory guidelines, among other things, offer clarity on the data needed to be generated by the owner of the vaccine to substantiate an efficacy claim. Such data may include the demonstration of specific antibodies to the target vaccine component(s) in vaccinated animals, and the results from challenge studies. Those specific antibodies need to be shown to exist, at the required level and for a defined time duration in offering likely protection to the vaccinated animals. Additionally, supportive data originating from field studies using vaccinated animals exposed to the target organisms are required. These data underpin the proposed vaccine product label information including claim, route of vaccination and vaccination regime.

However, in relation to vaccines targeting eukaryotic parasites in animals these similar regulatory requirements and supportive guidelines are in most cases unclear or missing. Within the European Union (EU), the European Pharmacopeia (EP) stipulates requirements that have relevance for example, anticoccidial vaccines for chickens (European Pharmacopeia, 2021), defining efficacy requirements. The EP offers quality standards and control of medicines. They provide a scientific basis for quality control during the entire life cycle of a product. They are mandatory on all EU States Parties to the Convention. While these standards are helpful, the European Union Medicines Agency (the EU regulator of veterinary medicines and biologics) does not appear to provide clear guidance across the range of eukaryotic organisms for the types of efficacy data required for vaccines.

Traditionally regulatory guidance on eukaryotic parasites have focused on chemical/drug-based products (antiparasiticides) with the supporting guidelines targeting quantitative percentage efficacy standards needed to obtain a specific product label claim.

Interrogating the few existing commercial vaccines targeting eukaryotic parasites offers insights into how regulators today are dealing with such product applications even though clear regulatory guidance does not appear to be in the public domain. Here we take as an example the Barbervax vaccine (Barbervax, 2022) targeting a nematode parasite of sheep.

The Barbervax vaccine manufacturer presented to the Australian Pesticides and Veterinary Medicines Authority (APVMA) [Australian regulator] the (biological) scientific basis behind the vaccine, plus preliminary field study results and modeling to support the case for a combination of worm egg count reduction that was based on field study results, plus antibodies in the same vaccinated animals in relation to the results. In this case antibodies by themselves were not sufficient to demonstrate efficacy to the APVMA’s satisfaction, as the relationship with efficacy was not quantitative or consistent for this particular vaccine. However, APVMA accepted that the results with this candidate vaccine was different from a classical anthelmintic product, i.e., reduction of worm burdens was not a key criterion and as the claim for this vaccine is primarily about a reduction in worm egg pasture contamination, thus demonstrating an effect of reducing worm egg counts was acceptable. Data generated with the Barbervax vaccine demonstrated a reasonable reduction in *Haemonchus* worm egg count of 75 - 90% compared to unvaccinated control animals, after the third vaccination. Additionally, modeling demonstrated a significant downstream epidemiological effect in reducing pasture contamination with worm eggs. The product claim for reducing disease due to *Haemonchus* worm infection was accepted by the APVMA as a consequence.

For this unique vaccine product, it appears a mix of regulatory requirements that already exist for vaccines targeting prokaryotic organisms plus existing requirements relating to anthelmintic product efficacy were imposed on the Barbervax product applicant by the APVMA.

While this is insightful, we still have no clear regulatory guidance in the public domain on how to progress development of upcoming vaccines targeting eukaryotic parasites. At present it appears to be the case that a vaccine developer should approach the respective regulator and seek unique counsel on a case- by -case basis.

Successful commercial vaccines targeting eukaryotic parasites of livestock in the future would likely form part of a multidisciplinary, holistic focus integrating chemotherapy, grazing management practices, biological control methods and host animal genetic resistance to parasites (Sharma et al., 2015; Claerebout and Geldhof, 2020). With this in mind it is important that clear direction is offered up by regulators on data requirements for vaccine products in such situations.

Is there a role for the International Cooperation for the Harmonization of Technical Requirements for the Registration of Veterinary Medicinal Products (VICH) in facilitating the release of efficacy requirements and guidelines relating to vaccines targeting eukaryotic parasites? VICH’s remit, among other things, is to deliver on harmonizing existing regulatory requirements of specific countries and regions for defined product types. VICH’s present remit does not permit it to consider regulatory issues relating to ectoparasites/ectoparasiticides. Its present activity in relation to efficacy of products targeting eukaryotic parasites is principally limited to anthelmintics based on chemical formulations – not vaccines. So, as no clear regulatory requirements for the vaccine topic at issue here exist in the public domain for each country/region that is a VICH member, it may be difficult for VICH to initiate harmonization.

The World Association for the Advancement of Veterinary Parasitology has traditionally offered guidelines on efficacy evaluation of antiparasiticides and these have subsequently become standard reference texts within the veterinary pharmaceutical industry and with some regulators. It may be that this association can repeat that for vaccines targeting eukaryotic parasites. However, these guidelines would not have any mandate unless adopted or approved by regulatory authorities.

So, when it comes to eukaryotic parasite vaccine products for animals and fish the current lack of regulatory direction in the public domain is a clear commercial hindrance to R&D programs of veterinary biological/pharmaceutical companies.

## Discussion

Consideration needs to be given by regulators on the future direction they will offer to the veterinary/biological pharmaceutical industry for the generation of efficacy data for vaccines targeting eukaryotic parasites. This direction must include definitions for “efficacy”; direction on what type of data needs to be generated to support a product label claim; and importantly a decision on whether regulatory direction will primarily focus on the host; on the parasite or on the technology behind the vaccine itself. It probably will need to be a combination of these three components and perhaps others.

Who will be the lead in filling this existing regulatory void?

## Author contributions

Both authors contributed equally to the opinion piece in content, analysis and drafting of the text.

## Conflict of interest

Both authors are the owner and director of their respective companies.

The authors declare that the research was conducted in the absence of any commercial or financial relationships that could be construed as a potential conflict of interest.

## Publisher’s note

All claims expressed in this article are solely those of the authors and do not necessarily represent those of their affiliated organizations, or those of the publisher, the editors and the reviewers. Any product that may be evaluated in this article, or claim that may be made by its manufacturer, is not guaranteed or endorsed by the publisher.
